# The function of previously unappreciated exerkines secreted by muscle in regulation of neurodegenerative diseases

**DOI:** 10.3389/fnmol.2023.1305208

**Published:** 2024-01-05

**Authors:** Xuepeng Bian, Qian Wang, Yibing Wang, Shujie Lou

**Affiliations:** ^1^School of Exercise and Health, Shanghai University of Sport, Shanghai, China; ^2^Institute for Health and Sport, Victoria University, Melbourne, VIC, Australia

**Keywords:** exercise, myokine, neurodegenerative disease, apelin, lactate, KYNA

## Abstract

The initiation and progression of neurodegenerative diseases (NDs), distinguished by compromised nervous system integrity, profoundly disrupt the quality of life of patients, concurrently exerting a considerable strain on both the economy and the social healthcare infrastructure. Exercise has demonstrated its potential as both an effective preventive intervention and a rehabilitation approach among the emerging therapeutics targeting NDs. As the largest secretory organ, skeletal muscle possesses the capacity to secrete myokines, and these myokines can partially improve the prognosis of NDs by mediating the muscle-brain axis. Besides the well-studied exerkines, which are secreted by skeletal muscle during exercise that pivotally exert their beneficial function, the physiological function of novel exerkines, e.g., apelin, kynurenic acid (KYNA), and lactate have been underappreciated previously. Herein, this review discusses the roles of these novel exerkines and their mechanisms in regulating the progression and improvement of NDs, especially the significance of their functions in improving NDs’ prognoses through exercise. Furthermore, several myokines with potential implications in ameliorating ND progression are proposed as the future direction for investigation. Elucidation of the function of exerkines secreted by skeletal muscle in the regulation of NDs advances the understanding of its pathogenesis and facilitates the development of therapeutics that intervene in these processes to cure NDs.

## 1 Introduction

With the aging of the population, the prevalence of aging-related brain health problems is soaring. Aging-related biomarkers (including β-galactosidase, tumor protein 53, cyclin-dependent kinase inhibitor 1A/2A, as well as the senescence-associated secretory phenotype) are also considered potential indicators within the domain of neurodegenerative diseases (NDs) (Hou et al., 2019). Previous research demonstrated that each of the nine hallmarks of aging (mitochondrial dysfunction, deregulated nutrient sensing, loss of proteostasis, cellular senescence, stem cell exhaustion, altered intercellular communication, genomic instability, telomere attrition, and epigenetic alterations) is linked to the development of NDs (Hou et al., 2019). NDs are further triggered by neuronal injury and subsequent demise, leading to the eventual compromise of a spectrum of neural functions encompassing motor, cognitive, and sensory domains. Chronic NDs, such as Alzheimer’s disease (AD), Parkinson’s disease (PD), Huntington’s disease (HD), and amyotrophic lateral sclerosis (ALS), are common in the elderly population and reduce the quality of life and whole lifespan. By 2050, there will be more than 2 billion people over 60 years old and more than 100 million patients with ND globally (Harper, 2014; Bloom et al., 2015; De la Rosa et al., 2020). Consequently, the onset and progression of ND evokes a substantial burden on the social economy.

The pathogenesis of NDs involves multiple key stages and hallmarks, including proteotoxic stress, autophagy/lysosomal abnormalities, oxidative stress, and neuroinflammation (Redmann et al., 2016; Su and Dai, 2016; Kwon and Koh, 2020; Abdelhamid and Nagano, 2023). In the pre-onset stages of NDs, multiple physiological abnormalities (pathological protein aggregation, synaptic and neuronal network dysfunction, protein homeostasis abnormalities, cellular cytoskeleton abnormalities, altered energy metabolism, DNA and RNA defects, inflammation, and neuronal cell death) frequently occur, leading to heterogeneous diagnostic criteria for different diseases (Azam et al., 2021). For example, positive β-amyloid (Aβ) and tau proteins are important diagnostic criteria before and during the onset of AD, but they do not necessarily serve as biomarkers at other stages (Dubois et al., 2021). The detection of cerebrospinal fluid (CSF) and serum biomarkers, including a-synuclein and PD protein 7, is still under development (Chahine and Stern, 2011; Kalia, 2019; Mollenhauer and von Arnim, 2022).

Current therapeutics of ND mainly depend have significant limitations in the efficacy and have adverse effects, due to the complex pathogenesis and clinical manifestations. Most target therapy are palliative and provide temporary relief for certain symptoms with minimal effect on the overall disease progression. These drugs often have noticeable adverse effects that impede their application in the treatment of NDs (Table 1).

**TABLE 1:** Therapeutic targets and side effects of ND drugs.

NDs	Main targets	Representative drugs	Adverse effect
AD	APP	Lecanemab, aducanumab-avwa	ARIA, headache, worsening confusion, dizziness, visual disturbances, nausea, and seizures (Withington and Turner, 2022; Soderberg et al., 2023)
	AChE	Donepezil, memantine, galantamine, rivastigmine	Bradycardia, electrocardiogram PR prolongation, GI adverse events, CNS palsy, nausea, vomiting, diarrhoea, anorexia, and weight loss (Igeta et al., 2013; Zemek et al., 2014)
	NMDAR	Bupropion/dextromethorphan, memantine	Dizziness, nausea, headache, somnolence, and dry mouth (Iosifescu et al., 2022)
PD	DRDs	Levodopa/carbidopa/entacapone, apomorphine, HP-200	Diarrhea, nausea, dizziness, somnolence, hypotonia, administration site reactions, and GI adverse events (Author, 1995; Lew et al., 2011; Carbone et al., 2019)
	D2 receptor	Rotigotine, pramipexole, ropinirole, talipexole, pergolide mesylate, tiapride, lisuride maleate	Hypotension, nausea, vomiting, drowsiness, tachycardia, impulse control disorder, sleep attacks (Alonso Canovas et al., 2014; Moretti et al., 2014)
	mAChRs	Trihexyphenidyl, bornaprine, mazaticol hydrochloride hydrate, tropatepine	High fever, SIADH, dry mouth, jitteriness, stomatitis, blurred vision, and forgetfulness (Jabbari et al., 1989; Zhao et al., 2020; Nigam et al., 2021)
	DDC	Levodopa/carbidopa/entacapone, carbidopa/melevodopa, levodopa/benserazide	Diarrhea, nausea, and dizziness (Lew et al., 2011)
	COMT	Levodopa/carbidopa/entacapone, opicapone, entacapone, tolcapone	Diarrhea, nausea, dizziness, dyskinesia, dry mouth, constipation, and hepatotoxicity (Leegwater-Kim and Waters, 2006; Lew et al., 2011; Feldman and Margolesky, 2023)
	MAO-B	Safinamide, rasagiline, selegiline	Insomnia, Confusion, Nausea, Dizziness, malaise, hypertension, orthostatic hypotension, headache, vomiting, impaired liver function, joint pain, hallucinations, anxiety, rash, back pain, transient mild dyskinesia (Dezsi and Vecsei, 2017)
HD	VMAT2	Valbenazine, deutetrabenazine, tetrabenazine	Somnolence, dry mouth, akathisia, headache, urinary retention, insomnia (Arya et al., 2019)
ALS	BAX	Tauroursodeoxycholic acid/sodium phenylbutyrate	Menstrual dysfunction, somnolence, constipation, diarrhea, nausea, and abdominal bloating (Sun et al., 2023)
	HDAC	Tauroursodeoxycholic acid/sodium phenylbutyrate	Menstrual dysfunction, somnolence, constipation, diarrhea, nausea, and abdominal bloating (Sun et al., 2023)
	SCNA	Riluzole, edaravone	Asthenia, gastrointestinal, elevated liver enzyme levels, urine glucose (Jaiswal, 2019)

APP, amyloid precursor protein; AchE, acetylcholinesterase; NMDAR, *N*-methyl-D-aspartate receptor; DRDs, dopamine receptor families; D2 receptor, dopamine D2 type receptor; mAChRs, muscarinic acetylcholine receptors; DDC, DOPA decarboxylase; COMT, catechol-O-methyltransferase; MAO-B, monoamine oxidase B; VMAT2, vesicular monoamine transporter 2; BAX, b-cell lymphoma-2 associated X protein; HDAC, histone deacetylase; SCNA, sodium channel α-subunit.

**FIGURE 1:**
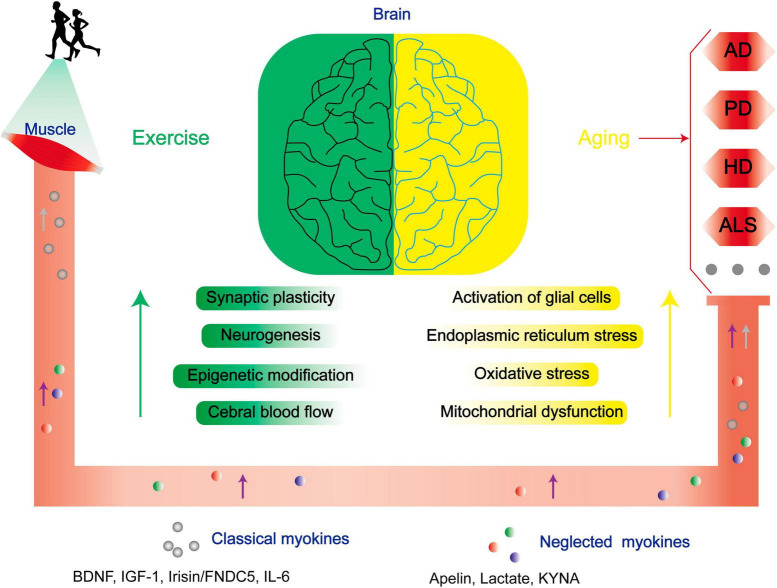
Exercise improves the prognoses of patients with NDs through multiple mechanisms. As aging progresses, there is a conspicuous escalation in the susceptibility to NDs. Physical exercise enhances synaptic plasticity, neurogenesis, epigenetic modifications, and central cerebral blood flow, and mitigates neurodegenerative risk factors linked to age-related glial cell activation, endoplasmic reticulum stress, oxidative stress, and mitochondrial dysfunction via modulation of both classical and neglected myokines. BDNF, brain-derived neurotrophic factor; IGF-1, insulin-like growth factor-1; CTSB, cathepsin B; FNDC5, fibronectin type III domain-containing protein 5; IL-6, interleukin 6.

In addition to therapeutics against NDs, exercise significantly ameliorate NDs in the central nervous system (CNS) by dampening key pathogenic pathways in ND (Figure 1). For example, exercise diminishes inflammation by preventing central microglial activation (Mee-Inta et al., 2019). Simultaneously, it employs a multitude of molecular pathways. It triggers the activation of the Nrf2-Keap1 system, orchestrating the transcription of genes linked to oxidative processes. Furthermore, it enhances BDNF expression, thereby regulating specific oxidative enzymes, while elevating lactate levels to facilitate the efficient clearance of superoxide anions and hydroxyl radicals. These coordinated mechanisms augment redox-sensitive signal transduction, fortify antioxidant capacity, and mitigate the pathological consequences of various conditions, including Alzheimer’s disease (AD) (Quan et al., 2020). In addition, exercise also involves in the modulation of neuronal survival and plasticity, neurogenesis, epigenetic modification, regulation of cerebral blood flow, angiogenesis, autophagy, and endoplasmic reticulum stress (ERS) (Sujkowski et al., 2022).

**FIGURE 2:**
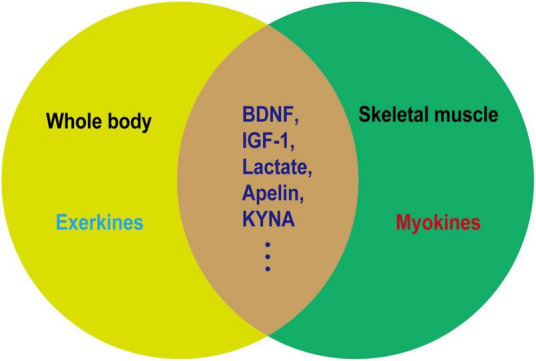
Relationship between exerkines and myokines. Exerkines are factors that are induced by exercise in various organs. Myokines are factors secreted by muscles and not necessarily induced by exercise. As depicted in the figure, a noticeable overlap exists between exerkines and myokines. The overlapping segment represents factors induced in muscles as a result of exercise, which may serve as the molecular foundation for elucidating the health-promoting effects of physical activity.

**FIGURE 3:**
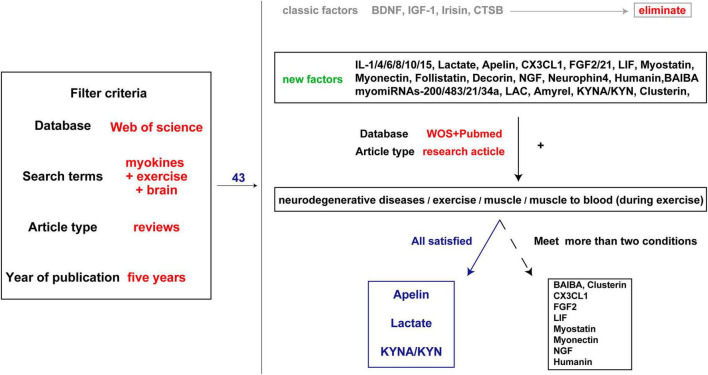
Literature screening process. The first step is to search all the brain and exercise-related muscle factors involved in the review published over the past 5 years and exclude the well-studied factors such as BDNF. The second step is to search the factors and keywords such as ND, exercise, and muscle, and find evidence of muscle secretion into the blood. Three factors with genetic evidence and several factors with partial evidence were identified.

The beneficial effect of exercise on promoting brain health is closely associated with the secreted factors known as exerkines. These exerkines are, in part, secreted by skeletal muscles and belong to the category of myokines, including brain-derived neurotrophic factor (BDNF), insulin-like growth factor-1 (IGF-1), cathepsin B (CTSB), irisin/fibronectin type III domain-containing protein 5 (FNDC5), and interleukin 6 (IL-6) (Febbraio et al., 2004; Lee et al., 2021). As an illustration, IGF-1 and FGF21 have been demonstrated to mitigate apoptosis by activating protein kinase B (Akt) in animal models of AD and PD, as well as *in vitro* cellular models employing SH-SY5Y and PC12 cells (Wang et al., 2017; Zhang et al., 2017; Chen et al., 2019; Fang et al., 2020). Meanwhile, irisin/FNDC5/CTSB can promote adult nerve regeneration through BDNF-mediated signaling pathways (Wrann et al., 2013; Moon et al., 2016); Besides, BDNF inhibits the production of Aβ (Rohe et al., 2009). The effects of these exerkines encompass a range of benefits, such as improving mitochondrial function, reducing oxidative damage, maintaining protein homeostasis, and promoting synaptic plasticity (Lee et al., 2021). Therefore, exploring the performance of different exercise factors in patients with NDs is particularly important to find new therapeutic targets and strategies for intervention in NDs. In this review, we focused on the role of some previously unappreciated factors secreted by skeletal muscles in regulating the progression of NDs. Moreover, the secreted factors induced during exercise that are not associated with skeletal muscle are excluded (Figures 2, 3).

## 2 Previously unappreciated exerkines that may ameliorate NDs

Skeletal muscles secrete hundreds of factors during exercise (Catoire et al., 2014). In this review, we focus on the function of three previously unappreciated factors: Apelin, lactate, and kynurenic acid (KYNA). In physiological states, the secretion of these factors may not be predominantly attributed to skeletal muscle. For example, Apelin and its receptors are expressed in smooth muscles, lungs, and kidneys (Chapman et al., 2021), where they play a crucial role in modulating vascular tone (Mughal and O’Rourke, 2018) and respiratory diseases (Yan et al., 2020). KYNA, predominantly produced through hepatic metabolism, has been the subject of increased scrutiny in the context of peripheral metabolic disorders (Zhen et al., 2022). Furthermore, lactate, traditionally associated with skeletal muscle metabolism, can also be secreted by a variety of cell types (Rabinowitz and Enerback, 2020). However, within the investigative framework of “exercise improve NDs,” the presence of these factors has been discerned within the secretome of skeletal muscles. This chapter offers a comprehensive overview of the origins, regulatory mechanisms, and roles of these factors within the framework of NDs. Additionally, it delves into the intricate interplay between exercise, skeletal muscles, and NDs, with the aim of enhancing our comprehension of the intricate roles played by these factors in the context of NDs.

### 2.1 Apelin

#### 2.1.1 Apelin and its peripheral effects

Upon activation of G protein-coupled receptors (GPCRs), the release of cyclic adenosine monophosphate (cAMP) initiates changes in extracellular proton concentration, resulting in acidification. In Tatemoto et al. (1998) employed acidification promotion rates in CHO cells expressing the apelin receptor (APJ) to screen polypeptide concentrate from varied tissues. Apelin effectively binds to the APJ, which is found in the extract of bovine stomach tissue. Apelin can be hydrolyzed into peptides with different lengths, such as apelin-12, apelin-13, apelin-17, and apelin-36, among which apelin-13 is the most biologically APJ activator (Tatemoto et al., 1998).

The APJ is one of the GPCRs that is first discovered in 1993. Although it shares 30% amino acid homology with the angiotensin II receptor, it does not bind to angiotensin II (O’Dowd et al., 1993). Northern blot analysis and *in situ* hybridization studies showed the presence of APJ mRNA transcripts in the CNS, including the hippocampus, striatum, thalamus, cortex, and cerebellum (O’Dowd et al., 1993). These results indicate that the ligand of APJ may play an essential role in the CNS (Pitkin et al., 2010). Beyond its CNS-centric implications, the APJ extends its regulatory influence to diverse physiological domains, encompassing cardiovascular modulation, metabolic homeostasis, and inflammation regulation (Liu et al., 2020; Li et al., 2022; Wang et al., 2022).

Apelin, originally identified as an adipokine, exerts its influence through the apelin/APJ axis, participating in a multifaceted spectrum of processes involving vascular integrity, cardiac functionality, renal health, and perturbations in glucose metabolism (de Oliveira et al., 2022; Li et al., 2022). Apelin modulates cell survival and differentiation in bone marrow-derived mesenchymal stem cells (BMSCs) and other cell types by regulating processes such as autophagy, apoptosis, and inflammation, thereby exerting a protective role in bone-related disorders including osteoporosis, fracture healing, arthritis, and periprosthetic osteolysis (Gong et al., 2023). In addition, the expression of apelin in the uterus and ovary suggests its involvement in placental physiology and regulation of pregnancy (Dawid et al., 2021). Moreover, apelin is intricately involved in the oncogenic processes of diverse malignancies, encompassing gastric-esophageal carcinoma, colorectal carcinoma, pulmonary carcinoma, and breast carcinoma (Grinstead and Yoon, 2022).

#### 2.1.2 The role of apelin in NDs

Neuroinflammation, marked by glial cell activation and heightened pro-inflammatory mediators, frequently manifests in brain regions affected by Alzheimer’s disease pathology. This inflammatory cascade precipitates a reduction in diverse neuronal trophic factors, including BDNF, and triggers the accrual of neurotoxic agents, culminating in neurodegeneration (Min et al., 2015). In Streptozotocin (STZ)-induced AD rats model, the levels of apelin/APJ were decreased in the hippocampus, while intraventricular injection of apelin can reduce the activation of glial cells and the expressions of Interleukin-1β (IL-1β) and tumor necrosis factor α (TNF-α) in the hippocampus to alleviate neuronal damage (Luo et al., 2019). Simultaneously, Apelin-13 resulted in improved memory performance, as indicated by a higher discrimination index (DI) observed in the novel object recognition (NOR) test. Notably, this beneficial effect of apelin was attenuated when the tropomyosin-related kinase B (TrkB) receptor antagonist K252a was utilized for blocking (Luo et al., 2019). This finding suggests that the anti-inflammatory effect of apelin-13 may involve the BDNF/TrkB pathway. In the amyloid precursor protein swedish mutation/presenilin deletion mutation 9 (APPswe/PSΔE9) double transgenic AD model study, the levels of peroxisome proliferator-activated receptor-gamma (PPARγ) co-activator-1alpha (PGC-1α)/PPARγ level were decreased (Chen et al., 2023). However, hippocampal injection of apelin-13 could dramatically reduce the levels of inflammatory factors, apoptosis, and oxidative stress-related proteins in the hippocampus. This treatment also results in a shortened escape latency during Morris water maze (MWM) learning in AD mice, accompanied by reduced time spent in the target quadrant and a decrease in the number of errors. Interestingly, the therapeutic effect of apelin-13 was diminished after co-injection with the PGC-1α inhibitor SR-18292 (Chen et al., 2023). Considering PGC-1α involves in the oxidative stress, inflammation, and apoptosis by regulating mitochondrial function, it is reasonable to suggest that apelin-13 ultimately provides neuroprotection by its targeting PGC-1α and mitochondrial function (Tang, 2016; Fontecha-Barriuso et al., 2020; Rius-Perez et al., 2020).

Nonetheless, discrepancies arise across studies. For example, in a rat model induced with neuroinflammation and cognitive decline via intraventricular lipopolysaccharide (LPS) injection, apelin-13 was found to decrease IL-1β and TNF-α levels. Additionally, it enhanced recognition rates in the NOR experiment and improved spontaneous alternation rates in the Y maze test (Hu et al., 2022). Meanwhile, apelin-13 elevates the level of glucocorticoid receptor (GR) and reduced its nuclear translocation (Hu et al., 2022). This role of apelin-13 may be related to its regulation of FK506-binding protein 51 (FKBP5), an auxiliary chaperone of GR that modulates GR sensitivity and its nuclear translocation (Aminyavari et al., 2019b). *In vitro*, stimulation of M1-type polarized N9 microglia by LPS resulted in the release of IL-6, activation of the downstream signal transducer and activator of transcription 3 (STAT3) pathway, whereas apelin-13 blocked the LPS-induced phosphorylation of STAT3 in polarized M1-type N9 microglia (Zhou et al., 2019). These findings showed that apelin is beneficial to ND treatment through improvement in multiple aspects.

In another representative ND, PD, the mechanism of apelin differs from that in AD. Apelin mainly targets ERS and autophagy function, both of which play a role in the pathogenesis of PD. In a PD mice model induced by 1-methyl-4-phenyl-1,2,3,6-tetrahydropyridine (MPTP), apelin-13 prolonged the latency time in the pole test and alleviated motor behavioral deficits, inhibited dopaminergic neurodegeneration, reduced α-synuclein expression in the substantia nigra pars compacta, and enhanced the level of autophagy in this region (Zhu et al., 2019b). Moreover, a study on SH-SY5Y cells reveals that apelin-13-induced autophagy involves the AMPK/mTOR/ULK1 signaling pathway (Chen et al., 2020). Additionally, apelin-13 reduces the activation of ERS-related signaling pathway IRE1α/XBP1/CHOP triggered by abnormal accumulation of α-synuclein and inhibits ERS-related apoptosis (Jiang et al., 2018; Zhu et al., 2019b).

Parkinson’s disease is not only characterized by dopaminergic neurodegeneration but is also associated with abnormal firing patterns in neural pathways (Matthews et al., 2023). Dysfunction in synaptic plasticity plays a crucial role in the development of motor complications associated with PD (Speranza et al., 2021). In the progression of PD, there is a decrease in the expression of synaptic plasticity-related proteins, including postsynaptic density protein 95 (PSD95), neurexin-1, and neurigin in striatal middle spine neurons. However, central administration of apelin-13 reversed this downregulation and significantly improved motor impairments, including enhanced balance, reduced rigidity, and increased locomotor distance. This suggests the potential of apelin-13 in modulating striatal neuronal degeneration, thereby effectively enhancing synaptic structural plasticity and functional plasticity (Haghparast et al., 2019; Liu et al., 2022). The *In vitro* neuroprotective effect of apelin may be achieved through anti-oxidation and anti-apoptosis in 6-hydroxydopamine-induced neurocytotoxicity in SH-SY5Y (Pouresmaeili-Babaki et al., 2018).

In an ALS model, the deficiency of apelin exhibited an earlier decline in motor performance in the rotarod test and an earlier onset of hind limb tremors, thereby accelerating the progression of ALS. Conversely, supplementation of apelin enhanced the protective effect of vascular endothelial growth factor (VEGF) against H_2_O_2_-induced primary neuron death, but the underlying mechanisms of these effects are not fully understood (Kasai et al., 2011). It is speculated that in ALS, apelin may partially improve the disease process through anti-inflammatory and other mechanisms, possibly due to the activation of microglia in response to apelin deficiency (Zhou et al., 2019). Although there may be some similarities in the pathogenesis of different NDs, the role of apelin is not consistent and even exert protective effects through opposite mechanisms. For instance, the neuroprotective effect of apelin-13 may be related to its ability to inhibit autophagy and apoptosis through the mTOR signaling pathway (Aminyavari et al., 2019a). But this finding contrasts with the role of apelin-13 in increasing autophagy levels observed in PD (Chen et al., 2020). In addition, besides the most active fragment apelin-13, other apelin fragments may have specific neuroprotective effects. In follow-up studies, apelin-36 exerts neuroprotective effects similar to apelin-13 in PD models (Zhu et al., 2019a,c). Furthermore, an *in vitro* study finds that apelin-36 exerts a cytoprotective influence on MPP^+^-induced SH-SY5Y cytotoxicity through the PI3K/Akt/mTOR autophagy pathway (Zhu et al., 2020).

#### 2.1.3 Contribution of myogenic apelin and effects of exercise

Tead1 is intricately linked to vital biological processes, encompassing cell proliferation, differentiation, and tissue development (Zhou et al., 2016; Liu et al., 2023). Its interaction with the regulatory domains of the apelin gene yields the potential to modulate the transcription and ensuing expression of apelin (Lee et al., 2022). Employing siRNA to abrogate Tead1 within skeletal muscle engenders a notable 50% elevation in apelin mRNA levels, coupled with a concurrent 24% augmentation in apelin protein content within muscle cells (Lee et al., 2022). Conversely, overexpression of Tead1 causes a reduction in the levels of apelin mRNA and peptide in skeletal muscle, accompanied by a decrease in the level of apelin in serum (Lee et al., 2022). Notably, the level of apelin in plasma gradually decreases with age, and this decline is associated with age-related muscle loss and osteoporosis (Vinel et al., 2018). Importantly, immunohistochemistry and liquid chromatography-mass spectrometry (LC-MS) techniques demonstrate that decreased plasma apelin level is specifically correlated with the loss of apelin mRNA level in skeletal muscle (Vinel et al., 2018).

**FIGURE 4:**
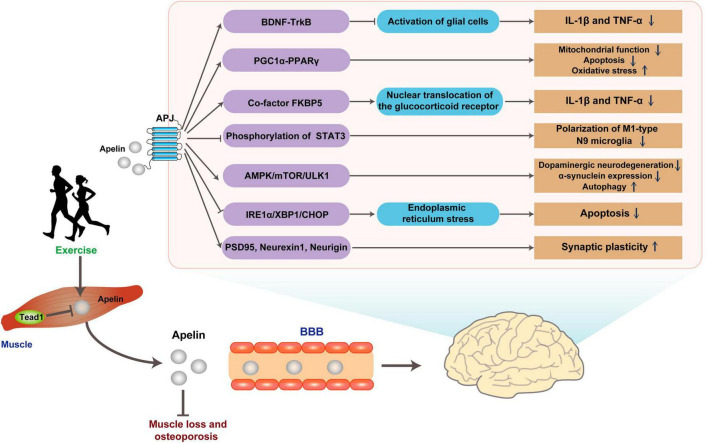
Possible pathways for exercise to improve brain health through Apelin. Apelin released in response to exercise binds the G protein-coupled receptor APJ, improving ND pathology through multiple pathways. BBB, blood-brain barrier.

Both simulated muscle contraction and exercise can promote the release of apelin in skeletal muscle, as demonstrated by previous studies (Vinel et al., 2018; Kwak et al., 2019; Lee et al., 2022). Muscle cells sourced from young donors demonstrated a rapid elevation of apelin levels in the culture supernatant within 5 min post-electrical stimulation, sustaining this increase for a duration exceeding 60 min. Conversely, cells derived from elderly donors necessitated a 30-min stimulation period to manifest a comparable augmentation (Vinel et al., 2018). Similarly, *in vivo* exercise study shows a comparable pattern. Acute exercise leads to an increase in the level of apelin in the plasma of young mice, while the immune response to apelin in the plasma of aged mice is attenuated. However, medium and long-term exercise can compensate for the age-related decline in the secretion of apelin in the skeletal muscle (Vinel et al., 2018; Figure 4 and Table 2).

**TABLE 2:** The relationship between exercise forms and apelin levels.

Exerkines	Exercise	Intensity and duration	Sample	Subject	Modulation
Apelin	Aerobic exercise	Single	Blood	Male human (Son et al., 2019; Ligetvari et al., 2023) Male rats (Kon et al., 2022)	↑
				Male human (Dogru et al., 2021; Tee et al., 2023) Human (Waller et al., 2019)	–
			Muscle, adipose tissue, heart, or liver	Male rats (Kon et al., 2022)	–
		Short-term (within 8 weeks)	Blood	Pregnant female mice (Son et al., 2022)	↑
				Male rats (Son et al., 2017)	–
			Muscle	Male rats (Son et al., 2017)	↑
				Mice (Ji et al., 2017)	–
			Heart	Male rats (Ashraf and Roshan, 2012)	↑
		Long-term (over 8 weeks)	Blood	Adolescent human (Dundar et al., 2021)	↑
				Male rats (Delavar and Heidarianpour, 2016) Middle-aged obese human (Sheibani et al., 2012; Galedari et al., 2017; Otero-Diaz et al., 2018; Dodevska et al., 2021)	↓
				Male rats (Kazemi and Zahediasl, 2018)	–
	Resistance exercise	Single	Blood	Male human (Bilski et al., 2016; Fortunato et al., 2018; Kon and Suzuki, 2022)	↑
		Short-term (within 8 weeks)	Blood	Middle-aged male human (Akyüz et al., 2021)	↓
		Long-term (over 8 weeks)	Blood	Postmenopausal women (Ghanbari-Niaki et al., 2020)	↑
				Middle-aged male human (Galedari et al., 2017)	↓
	HIIT	Single	Blood	Male human (Kon et al., 2021)	↑
		Long term (over 8 weeks)	Blood	Middle-aged male human (Galedari et al., 2017)	↓
	Daily physical activity	Visceral and subcutaneous adipose tissue		Human (Mahmoodi et al., 2019)	↑

HIIT, high-intensity interval training.

### 2.2 Lactate

Since its discovery in 1780, lactate is used to conceived as a simple metabolic waste product and an anoxic product (Opie, 1978; Siess, 1980). However, in the 1980s, the understanding of lactate function was revolutionized with the lactate shuttle proposed by Brooks (1986). Lactate plays a vital role as an essential energy metabolic substrate and serves as a critical precursor for gluconeogenesis (Brooks, 2020; Rabinowitz and Enerback, 2020). In human tissues and blood, the predominant form of lactate is L (+) lactate (Ferguson et al., 2018). Under resting conditions, the serum concentration of L (+) lactate is about 1 mM, and the lactate concentration in CNS is 0.1–1 mM, which is produced by astrocytes and then transferred to neurons (Ferguson et al., 2018). However, during a short period of maximum-intensity exercise, the level of lactate in serum rises quickly with concentrations exceeding 15 mM, and peaking at 20-30 mM (Ferguson et al., 2018). During exercise, a portion of lactate in blood is transported to the liver, where it is utilized as the starting material for gluconeogenesis to provide energy for exercise. Alternatively, lactate can enter the amino acid metabolism pathway through transaminase. Additional lactate is conveyed to the brain via monocarboxylate transporters (MCTs) (Iwanaga and Kishimoto, 2015). Consequently, the cerebral lactate level can elevate to 2 mM (Bergersen, 2015; Mosienko et al., 2015).

#### 2.2.1 The role of energy supply

Exercise substantially elevates cerebral lactate levels through two distinct mechanisms. Firstly, exercise directly augments lactate levels in the bloodstream, subsequently facilitating its transportation to astrocytes and neurons in the brain via monocarboxylate transporters (MCTs) (Bergersen, 2015). Secondly, exercise improves neuronal activity by releasing the excitatory transmitter glutamate, which stimulates glycolysis in astrocytes. This glycolysis process allows for the removal of lactate, which is subsequently taken up by neurons and utilized in their metabolic processes (Vardjan et al., 2018). Previous study shows that the knockout of astrocyte MCT1/4 or neuronal MCT2 by antisense oligodeoxynucleotides leads to significant memory retention impairments observed in the Y-maze and MWM tests in rats. Interestingly, the memory impairment caused by MCT1/4 knockout can be improved by injection through the hippocampal CA1 region, but no difference in the memory impairment caused by MCT2 knockout (Suzuki et al., 2011). Moreover, the inhibition of astrocyte glycogen metabolism by diaminobenzidine (DAB) can reduce long-term potentiation (LTP) and impair memory consolidation in the hippocampus (Suzuki et al., 2011). In contrast, the co-administration of DAB and lactate oppose this outcome, as the reduction in astrocytic MCT1/4 levels nullifies the beneficial influences of lactate (Suzuki et al., 2011). Additionally, 2-h of moderate-intensity treadmill exercise increases the levels of lactate and MCT1/2 in the cerebral cortex and hippocampus of rats. Moreover, the level of MCT2 is positively correlated to the levels of exercise-regulated neurons BDNF/TrkB (Takimoto and Hamada, 1985). Furthermore, a 4-week moderate-intensity treadmill exercise elevates the level of MCT2 in the hippocampus and improves spatial memory performance in diabetic rats (Shima et al., 2017).

#### 2.2.2 Role of lactate as a signaling molecule

Apart from its function as an energy substrate, lactate plays an important role in influencing learning and memory via other mechanisms. Lactate is a ligand for the hydroxycarboxylic acid receptor 1 (HCAR1), which belongs to the Gi-coupled receptor type, also known as GPR81. In the brain, GPR81 is mainly localized on the membrane of excitatory synapses, primarily in the postsynaptic membrane, followed by vascular endothelial cells and astrocyte terminals (Lauritzen et al., 2014). It is known that the Giα subunit and βγ subunit of GPCRs can regulate tissue and cell metabolism by activating ERK1/2 (May and Hill, 2008). Activation of ERK1/2 affects the transcriptional expression of synaptic plasticity-related genes, such as immediate early genes (IEGs), BDNF, and vascular growth factor (VGF), and ultimately increases neuronal synaptic plasticity, thereby contributing to improved learning and memory function (Yang et al., 2014). Brain lactate also serves as a signaling molecule that can activate NMDARs containing the subunit 2B (NR2B) domain, thereby enhancing calcium (Ca^2+^) influx (Zhang et al., 2016; Jourdain et al., 2018). The primary mechanism involves the transport of lactate to neurons through MCT2, where it is converted to pyruvate under lactate dehydrogenase 1. During this process, NAD^+^ is reduced to NADH and activates NMADRs, evoking a Ca^2+^ influx through the activation of calcium/calmodulin-dependent protein kinase 2/4 (CaMKII/IV) (Bickler et al., 2009). The Ca^2+^ influx triggers downstream signaling pathways involving cAMP response element binding protein (CREB), leading to increased levels of synaptic plasticity-related transcription factors and proteins (Zhao et al., 2007).

The increase in blood lactate level can activate ERK and Akt through GPR81, which is caused by high-intensity interval training (HIIT). This activation leads to the upregulation of VEGF and then promotes microangiogenesis in the hippocampus of mice (Morland et al., 2017). Further study reveals that a 7-week HIIT or subcutaneous injection of lactate could activate Akt through GPR81 and promote neurogenesis in the subventricular zone (SVZ), as well as promotes neurogenesis in the subgranular zone (SGZ) of the hippocampal dentate gyrus (Lambertus et al., 2021). Interestingly, lactate injection or Gpr81 knockout does not affect hippocampal neurogenesis (Lambertus et al., 2021). Conversely, another study shows that intravenous infusion of lactate for 7 weeks increases the number of new mature neurons in the hippocampus of mice, independent of GPR81 activation (Lev-Vachnish et al., 2019). Furthermore, behavioral tests such as the radial arm water maze (RAWM) and Barnes maze indicated that there were no significant differences among the groups, and lactate treatment did not affect hippocampus-dependent spatial learning (Lev-Vachnish et al., 2019). Therefore, the mechanism by which exercise-induced increase in lactate/GPR81 affects hippocampal synaptic plasticity is still inconclusive and needs further investigation.

#### 2.2.3 Dual identity in NDs

The roles of lactate in NDs are multifaceted, and its impact varies across different conditions. In patients with PD, the CSF lactate level increases with age, and notably, both early onset and late-onset PD show elevated lactate levels, despite variations in CSF biomarkers and clinical characteristics (Schirinzi et al., 2020). Similarly, in patients with remitting multiple sclerosis (MS), the level of lactate is elevated and inversely correlated with disease duration (Albanese et al., 2016). Moreover, the level of lactate is positively correlated with the levels of tau protein and neurofilament light chain in CSF, both of which are important biomarkers of neuropathy (Albanese et al., 2016). Furthermore, lactate level is increased in the CSF of AD patients, potentially related to impaired mitochondrial function (Liguori et al., 2015). These findings suggest a potential link between lactate dysregulation and ND’s pathophysiology. Paradoxically, the level of lactate in CSF is decreased in patients with AD and frontotemporal dementia (FTD). Notably, the level of lactate in the CSF is negatively correlated with the levels of total (t)-tau and phosphorylated (p)-tau proteins in patients with AD (Bonomi et al., 2021). In a PD toxicity model, lactate and pyruvate induce autophagy and mitophagy at non-toxic concentrations, providing cellular protection and reinstating mitochondrial function to counteract apoptosis and necrosis, as evidenced in both short-term and prolonged experiments (Fedotova et al., 2022). However, the ablation of the lactate dehydrogenase B (LDHB) gene promotes the conversion of lactate to pyruvate, leading to deficits in short-term working memory, spatial learning, and memory retention in mice. Pyruvate may induce brain oxidative stress, neuroinflammation, and neuronal apoptosis-mediated neurodegenerative processes by negatively regulating the p-AMPK/SIRT1/PGC-1α signaling pathway (Park et al., 2022).

**FIGURE 5:**
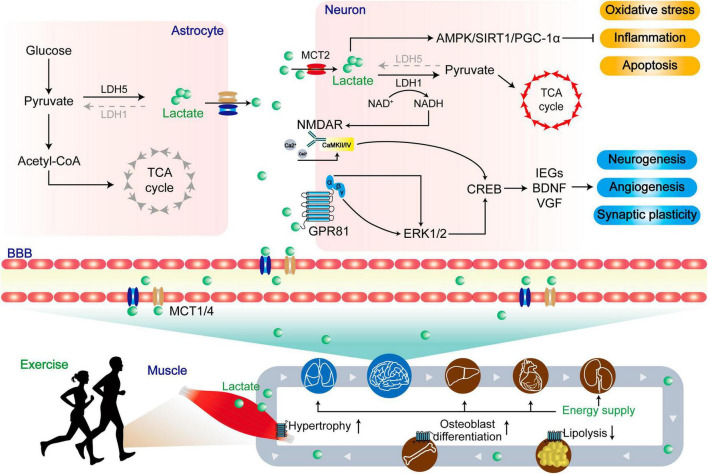
The dual role of lactate in central and peripheral functions. Lactate produced by skeletal muscle enters the periphery through MCTs to provide energy, resist lipolysis, promote osteoblast differentiation, and promote muscle hypertrophy. Upon entering the central region, lactate undergoes enzymatic conversion to pyruvate by lactate dehydrogenase, serving as a vital energy source within the tricarboxylic acid cycle (TCA). Concurrently, lactate activates the AMPK/SIRT1/PGC-1α pathways, leading to the mitigation of oxidative stress, inflammation, and apoptosis. In a distinct role, lactate functions as a signaling molecule. A portion of it engages with the lactate receptor GPR81, modulating ERK1/2 signaling, while another portion influences the glutamate receptor NMDAR via intracellular signaling. These integrated actions collectively foster neurogenesis, angiogenesis, and synaptic plasticity, ultimately enhancing the prognosis for patients afflicted by NDs.

The lactate receptor acts as a Gi-type G protein and inhibits adenylate cyclase upon activation. Forskolin, an adenylate cyclase agonist, reverses motor dysfunction, cognitive impairment, and excessive free radical production in HD animal models by activating the cAMP/PKA/CREB pathway (Mehan et al., 2017). Specifically, this is manifested as a significant reduction in the latency period of rats, an increase in the time spent in the target quadrant, along with improvements in grip strength, and a decrease in the number of slips during the beam traversal task. This finding suggests a potential negative role of GPR81 activation in HD, highlighting the complex involvement of lactate in brain function. Such complexity arises from the dual nature of lactate as both an energy supply and signaling molecule, as well as potential variations on the model of NDs. Notably, metabolomic analysis of postmortem frontal cortex samples from patients with AD and ALS reveals a discrepancy in lactate concentrations, with low levels in AD samples and higher levels in ALS samples (Botosoa et al., 2012; Figure 5).

### 2.3 Kynurenine (KYN) and KYNA

**FIGURE 6:**
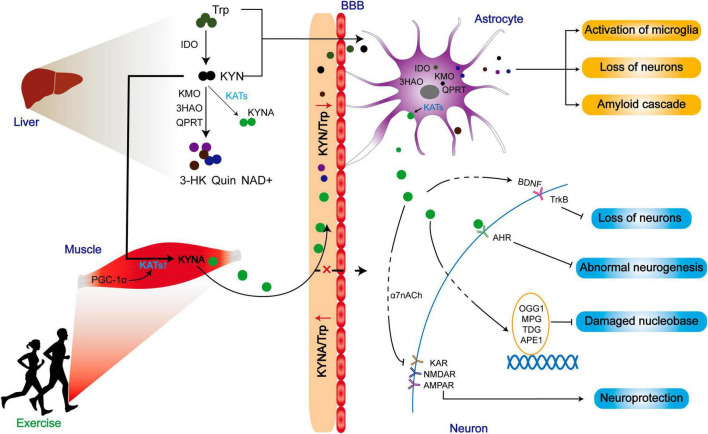
The improved prognosis of ND patients may be related to the reduction of central toxicity of KP by exercise. Exercise promotes the expression of KATs in skeletal muscle and converts peripheral KYN to KYNA. While peripherally generated KYNA may not exert positive effects by crossing the blood-brain barrier, the reduction of KYN/Trp indirectly lowers central KYN intake, thereby mitigating the central toxicity of the KP metabolites. Additionally, KATs within astrocytes can promote KYNA production, contributing to a range of neuroprotective effects.

Different from myokine, KYNA has a limited ability to cross the blood-brain barrier (BBB), but KYN can penetrate through the blood-brain barrier (Fukui et al., 1991). Surprisingly, skeletal muscle does not increase circulating KYNA into the brain for neuroprotection during exercise. Instead, it indirectly modulates the metabolic balance between KYN and KYNA in favor of KYNA, thereby reducing the central toxic effects of KYN and its downstream products (Figure 6).

#### 2.3.1 KYN pathway and central toxic effects

Tryptophan (Trp) is one of the essential amino acids for various physiological functions, which should be obtained through food intake (Comai et al., 2020). It is widely distributed in different tissues and organs throughout the body (Kaluzna-Czaplinska et al., 2019). Trp undergoes different secondary degradation pathways, including decarboxylation of tryptophan to tryptamine, transamination to indol-3-yl pyruvic acid, and hydroxylation to 5-hydroxytryptamine (5-HT), which contribute to its overall metabolic profile (Cervenka et al., 2017). The KYN pathway (KP) is responsible for the metabolism of more than 95% of tryptophan, which results in the production of various metabolites that exert different neurological effects. The function of the KP depends on the efficiency of distinct enzymes involved in the metabolic processes. Most tryptophan is metabolized by indoleamine 2,3-dioxygenase (IDO) to produce KYN, which is then excreted by the urine. However, when the synthesis of KYN exceeds renal clearance, KYN is hydroxylated to 3-hydroxykynurenine (3-HK) by kynurenine monooxygenase (KMO) and generates quinolinic acid (QUIN) in a non-enzymatic reaction, and is finally converted into NAD^+^ (Cervenka et al., 2017).

Neurotoxic effects in the CNS are attributed to metabolites of KP, which are increased with age (Kaiser et al., 2019; Hinkley et al., 2023). Specifically, 3-HK is identified as one of six potential blood biomarkers for PD by mass spectrometry analysis (Iwaoka et al., 2020). Furthermore, in the CSF of PD patients, the level of KYN and 3-HK metabolism are augmented, resulting in higher concentrations compared to the healthy control group (Klatt et al., 2021). Elevated levels of 3-HK and 3-hydroxy anthranilic acid enhance copper-induced toxicity in cultured rat astrocytes (Ramirez-Ortega et al., 2017). Additionally, QUIN, which is overproduced due to microglial activation, is implicated in the inflammatory pathogenesis of AD and the mechanism of ALS (Guillemin et al., 2003; Lee J. M. et al., 2017). The neurotoxic effects of these metabolites mainly depend on the key enzymes in the KP, with recent studies focusing on IDO and KMO (Thevandavakkam et al., 2010; Huang et al., 2020). Pertinently, the levels of IDO1 and KYN/Trp ratios are elevated in the serum and CSF of epileptic patients (Deng et al., 2021). In addition to these manifestations, the levels of pro-inflammatory cytokines, including IL-1β, IL-6, and TNF-α, are elevated in the serum and hippocampus of epilepsy model mice. Remarkably, the knockdown of IDO results in reduced KYN metabolites and neuronal loss in the mouse hippocampus, effectively suppressing seizures (Deng et al., 2021). These results suggest that KP plays a crucial role in regulating both innate and acquired immune responses, thereby reflecting its essential role in mediating the interface between immunity and the CNS (Mandi and Vecsei, 2012).

After intracerebroventricular injection of Aβ1-42 polypeptide, the level of IDO in the prefrontal cortex and hippocampus, as well as the levels of KYN and KYN/Trp are increased, which is accompanied by memory impairment, depression, and anxiety-like behaviors. However, the administration of 1-MT, an inhibitor of IDO, prevented these negative development (Souza et al., 2016).

Huntington’s disease is caused by an unstable expansion of glutamine encoded by the genetic codon CAG within exon 1 of the *HTT* gene, which encodes the huntingtin protein (Author, 1993). Interestingly, another rate-limiting enzyme in KP metabolism, KMO, is found to co-localize with HTT on the outer mitochondrial membrane of HEK293T cells, suggesting a potential interaction between KMO and HTT with the mitochondrial outer membrane (Swaih et al., 2022). Notably, in a Drosophila melanogaster HD model, the KMO inhibitors reduce neuronal loss and prevent neurodegeneration, suggesting the therapeutic potential of targeting KMO in HD (Zhang et al., 2019). Nevertheless, the biological significance of this interaction remains unclear and requires further elucidation to enhance comprehension of the pathological mechanisms underlying HD.

#### 2.3.2 KYNA and its neuroprotective effect on patients with NDs

Under certain conditions characterized by abundant tryptophan and KYN in circulation, KYN is converted to KYNA through the enzymatic activity of four kynurenine aminotransferases (KAT 1∼4, KATs), which are expressed in the brain, liver, and skeletal muscle (Cervenka et al., 2017). Notably, the synthesis of KYNA predominantly occurs in astrocytes, and its release is triggered by astrogliosis and inflammatory responses, both of which are hallmarks of AD (Maddison and Giorgini, 2015). Unlike other metabolites within the primary KP pathways, KYNA generally exerts neuroprotective effects in the CNS (Sas et al., 2018). Astrocytes exhibit neuroprotective properties by minimizing the production of QUIN and maximizing the synthesis of KYNA in the absence of macrophages or microglia. However, in the presence of these cells, astrocytes may manifest neurotoxicity (Guillemin et al., 2000, 2001). In the context of LPS-induced astrocyte activation and cytotoxicity, KYNA diminishes the activation of astrocytes while concurrently increasing the expression of anti-inflammatory M2 phenotype markers cluster of differentiation 163 (CD163) in microglia and significantly reducing pro-inflammatory M1 phenotype markers CD16 and CD32 (Majerova et al., 2022).

Kynurenic acid and its analog N-(2-(dimethylamino)ethyl)-3-(morpholinomethyl)-4-hydroxyquinoline-2-carboxamide (SZR104) significantly reduce the levels of inflammatory marker proteins C-X-C motif chemokine ligand 10 (CXCL10) and C-C chemokine receptor type 1 (CCR1) in microglia following LPS stimulation and alter the distribution and lysine methylation pattern of histone H3 in microglia (Szabo et al., 2022), impacting the activation or inhibition of genes related to immune cell differentiation and immune responses (Levinsky et al., 2022). These compounds also significantly inhibit the transition of hippocampal microglia from a resting state to an activated state and reduce the levels of IL-6 in the blood (Lajko et al., 2020), while suppressing the pro-inflammatory phenotype changes in neonatal rat forebrain microglia, with SZR104 exhibiting more pronounced effects (Szabo et al., 2023). Additionally, KYNA protects against QUIN-induced alterations in the cytoskeletal organization and cell morphology, partially inhibiting the QUIN-induced activation phenotype in microglia and preventing the upregulation of ionized calcium-binding adapter molecule 1 (Iba-1) induced by QUIN (Pierozan et al., 2018). These findings underscore the potential significance of KYNA and SZR104 in neuroprotection and the regulation of neuroinflammation. In *in vitro* models, KYNA reduced neuroinflammation induced by Aβ activation of BV-2 microglial cells, decreasing the release of pro-inflammatory cytokines TNF-α and IL-6 but also concurrently reducing Aβ phagocytosis (Steiner et al., 2014). These findings underscore the potential significance of KYNA in the context of AD, particularly in modulating Aβ-induced neuroinflammation, while also indicating the complexity and potential adverse effects of KYNA, such as reduced Aβ phagocytosis.

These neuroprotective properties are attributed to its ability to antagonize excitatory amino acid receptors (EAARs), such as *N*-methyl-D-aspartate receptor (NMDARs), α-amino-3-hydroxy-5-methyl-4-isoxazole propionic acid (AMPA) receptors, and kainate receptors (KARs). These effects are associated with the antagonism of the α7 nicotinic acetylcholine (α7nACh) receptor (Hilmas et al., 2001). After a traumatic brain injury (TBI), pretreatment with KYNA significantly reduced the response of hippocampal microglia and astrocytes, and effectively mitigated neuronal depolarization by inhibiting NMDAR and AMPAR (Suma et al., 2008). Whereas subsequent studies indicate that replicating these findings are proven challenging (Stone, 2007; Arnaiz-Cot et al., 2008; Mok et al., 2009; Zadori et al., 2016). Thus, the effects of KYNA on various tissues exhibit high diversity, depending on the type of receptors expressed within target cells. While the antagonism of NMDARs does not solely account for the observed effects, the stimulation of enkephalin expression and/or activity induced by low micromolar amounts of KYNA appears crucial for neuronal cell viability and survival in the human neuroblastoma SH-SY5Y cell line and primary cultures of mouse cortical neurons (Klein et al., 2013). Significantly, heightened levels of KYNA within the sheep central nervous system prompt the transcriptional upregulation of 8-oxoguanine glycosylase (OGG1), N-methylpurine DNA glycosylase (MPG), thymine DNA glycosylase (TDG), and AP-endonuclease (APE1) in the CA1 subregion of the hippocampus (Roszkowicz-Ostrowska et al., 2022). These enzymes play a pivotal role in mending oxidative and methylation-induced lesions within DNA (Iyama and Wilson, 2013). Furthermore, both high and low doses of KYNA promote increased levels of BDNF-TrkB in the hippocampus of sheep (Roszkowicz-Ostrowska et al., 2022). These findings highlight the essential roles played by KYNA in neuronal function and the development of degenerative diseases, potentially involving neuronal loss.

Patients afflicted with NDs exhibit a multifaceted and heterogeneous pattern in terms of KYNA levels within the cerebral milieu. Notably, a decremental trend in KYNA levels manifests in the CSF of select AD patients; however, conversely, escalated KYNA concentrations are discerned in distinct AD instances (Jacobs et al., 2019; Sorgdrager et al., 2019; van der Velpen et al., 2019). Intracerebroventricular injection of low-dose KYNA (0.5 μg/2 μL) enhances memory consolidation by increasing the escape latency in passive avoidance tests of mice, while high doses (40 μg/2 μL) impair memory performance (Klein et al., 2013). Importantly, a low dose (0.5 μg) of KYNA significantly extended the latency period for mice to enter the electric shock-associated dark zone, while conversely, a higher dose (40 μg) of KYNA significantly reduced the avoidance latency. This effect of low-dose KYNA is abolished upon the combination of 5-HT2 receptor antagonist cyproheptadine, α-adrenergic receptor antagonist phenoxybenzamine, opioid receptor antagonist naloxone, dopamine D2/D3/D4 receptor antagonist fluphenazine, β-adrenergic receptor antagonist propranolol, and muscarinic acetylcholine receptor antagonist atropine. This compellingly implies that the potential impact of KYNA might encompass intricate interplay within the serotonergic, adrenergic, dopaminergic, and opioidergic nervous systems (Martos et al., 2022).

In addition, KYNA also acts as an aryl hydrocarbon receptor (AHR) ligand. Accumulation of central amyloid in zebrafish decreased the level of KAT in CNS, leading to inhibition in the production of KYNA (Siddiqui et al., 2021). Interestingly, the compensatory mechanism of neurogenesis in the AD process is ablated upon the combination use of Aβ42 and AHR agonists. On the contrary, administration of AHR antagonists can promote the proliferation of neural stem cells (NSCs) (Siddiqui et al., 2021). This finding indicates that the KYNA/AHR signaling pathway inhibits neurogenesis under a physiological context, and its abnormal upregulation may further aggravate neuronal loss in AD progression. This study potentially explains the fluctuation of KYNA observed in CSF in AD-related studies, when the involvement of a compensatory mechanism is considered. Moreover, the reduction in the level of KYNA in patients with early AD might be a response to counteract the loss of NSCs caused by AD. It is suggested that low concentrations of KYNA can also exert positive effects through multiple systems, possibly contributing to this compensatory mechanism (Siddiqui et al., 2021). Conversely, high concentrations of KYNA may be attributed to the peripheral overload of KYN during the AD progression, which impairs the KP pathway in the central, resulting in an abnormal increase in KAT/KYNA ratio. Interestingly, the central elevation of KYNA appears to be specific to AD and not observed in other NDs such as FTD, ALS, or progressive supranuclear palsy (PSP) (Gonzalez-Sanchez et al., 2020).

#### 2.3.3 Effects of muscle dynamics on circulation and central KYNA levels during exercise

The positive effects of exercise are attributed to the secretion of KYNA. Exercise increases the level of KYNA in human skeletal muscles, plasma, and sweat (Schlittler et al., 2016; Allison et al., 2019; Saran et al., 2021; Shi et al., 2022). Since KYNA is unlike to cross the BBB, KYNA in the periphery may not directly and critically contribute to the neuroprotective effect. Instead, KYNA plays a regulatory role in the KP pathway by shifting the metabolism of the circulation of KYN toward KYNA (Fukui et al., 1991). By reducing the levels of circulating KYN and its subsequent metabolic products in circulation, KYNA can potentially mitigate the neurotoxic effects caused by excess central KYN.

Skeletal muscle as an important metabolic organ, regulates KAT function, which is influenced by PGC-1α transcription. Expression of PGC-1α leads to the upregulation of KAT1, 3, and 4 in skeletal muscle, thereby enhancing the metabolic pathway from peripheral KYN to KYNA, which diminishes the central inflammatory response associated with chronic mild stress (Agudelo et al., 2014). However, the loss of PGC-1α in skeletal muscle reduces KAT expression, rendering muscle susceptible to KYN toxicity (Agudelo et al., 2019). KAT is more highly expressed in oxidative muscle fibers than glycolytic muscle fibers. It is found that there is a higher mitochondrial abundance in oxidized muscle fibers, suggesting a close relationship between KYNA and mitochondria. Notably, this difference is not age-dependent (Wyckelsma et al., 2020). The level of four KAT mRNAs and proteins in skeletal muscle is, in comparison to individuals who do not exercise (Agudelo et al., 2014; Schlittler et al., 2016). Moreover, the level of KYNA in plasma instantaneously increased after exercise, surpassing the level of neurotoxic metabolites like QUIN. Of note, there are no changes observed in plasma KYNA and QUIN/KYNA ratios during high-intensity eccentric exercise (Schlittler et al., 2016). These results suggest that the exercise-induced pathway involving KAT/KYNA may be more dependent on prolonged aerobic exercise, which is closely linked to mitochondria activity. Although HIIT is generally considered an anaerobic-based exercise, it also effectively activates KAT/KYNA pathway (Javelle et al., 2021), but it seems to promote KYNA better than moderate-intensity chronic training (MICT). Notably, HIIT activates the KYN pathway to product KYNA more effectively than MICT in patients with MS (Joisten et al., 2021). In addition to exercise, drug administration or a ketogenic diet can increase the production of KYNA, and activates the PGC1α/PPARα signaling in skeletal muscle in normal or model mice (Pathak et al., 2022; Yin et al., 2022; Table 3).

**TABLE 3:** The relationship between exercise forms and KYNA levels.

Exerkines	Exercise	Intensity and duration	Sample	Subject	Modulation
KYNA (kynurenine pathway)	Aerobic exercise	Single	Hippocampus	Rats (Yamamoto et al., 2012)	↑KYNA ↑QUIN
		Short-term (within 8 weeks)	Hippocampus	Rats (Yamamoto et al., 2012)	↑KYNA ↑QUIN
			Muscle	Male human (Schlittler et al., 2016) OVX mice (Shi et al., 2022)	↑KATs
			Blood	Male human (Schlittler et al., 2016) Male mice (Lee K. J. et al., 2017) BDNF val knock-in mice (Ieraci et al., 2020) OVX mice (Shi et al., 2022)	↑KYNA
			Sweat	Aged 25 to 65 human with mild to moderate lower back pain (Saran et al., 2021)	↑KYNA ↓KYN
		Long term (over 8 weeks)	Blood	Patients with depression (Millischer et al., 2017)	–
	Resistance exercise	Long term (over 8 weeks)	Blood	Breast cancer patients (Zimmer et al., 2019)	↓KYN
	Stretching training	Long term (over 8 weeks)	Blood	Human (Javelle et al., 2021)	↓KYNA/QA ↓KYN/QA
	HIIT	Long term (over 8 weeks)	Blood	Human (Javelle et al., 2021)	↓KYNA/QA ↓KYN/QA

HIIT, high-intensity interval training; OVX, ovariectomized.

Recently, there have been numerous studies on the role of KP in the CNS upon exercise, which are mainly focusing on improving depression. In a murine model, IDO inhibitors prevent LPS-induced depression-like behavior, and injection of KYN can induce depression-like behavior in a dose-dependent manner (O’Connor et al., 2009). In parallel, KYNA can reverse this effect, thereby offering a potential therapeutic approach (Tanaka et al., 2020). Furthermore, 4 weeks of voluntary rolling wheel exercise can promote the level of KAT3 in murine skeletal muscle, thereby ameliorating KYN-induced depression-like behavior (Su et al., 2020). However, it is important that not all kinds of exercise support the antidepressant effects of KYN. For example, after 12 weeks of low to high-intensity aerobic exercise, moderate to mild depression patients experience improvement in mood and cardiovascular fitness, but neither KYN levels compared to KYNA levels nor their ratios in blood samples changed significantly (Millischer et al., 2017). Another study on the impact of branched-chain amino acids intake on plasma KYNA and skeletal muscle KAT4 levels revealed that changes in KYNA are unrelated to KYN metabolism, and the level of KAT4 in skeletal muscle is unaffected (Jonsson et al., 2022). These results suggest that other factors may easily offset the positive effect of exercise on skeletal muscle KP metabolism.

### 2.4 Similar effects in apelin, lactate, and KYNA

Based on previous studies, apelin, lactate, and KYNA share common characteristics in their function. Apelin exerts its effects through GPCRs (Tatemoto et al., 1998). Lactate not only engages with GPCRs but also functions as an energy substrate through its conversion into pyruvate (Suzuki et al., 2011; Morland et al., 2017). Peripheral KYNA plays a unique role in reducing central toxicity by modulating the conversion between KYN with KYNA (Agudelo et al., 2014). While these pathways exhibit distinct characteristics, they converge toward a shared objective of ameliorating NDs by mitigating a spectrum of adverse effects, encompassing anti-inflammatory and anti-apoptotic mechanisms.

The impact of these constructs on patients can exhibit variations influenced by the progression and nature of the disease. Consequently, it is imperative to incorporate data collected prior to, during, and following the onset of NDs within the studies, thereby enabling the acquisition of more robust evidence through cross-sectional analysis.

## 3 Other potential myokines

In addition, there are some overlooked exerkines with potential functions in the CNS or origin. The involvement of the chain of β-aminoisobutyric acid (BAIBA) and clusterin (CLU) in the exercise-muscle-ND axis is relatively direct, although the detailed mechanism remains elusive. In addition, we found some promising secreted factors during the screening process (Table 4).

**TABLE 4:** Other myokines secreted during exercise with function in ND not deciphered.

Myokines	Effects in NDs	Exercise stimulation	Expression in muscle	Muscle to blood (during exercise)
CX3CL1	√ (Subbarayan et al., 2022)	√ (Njerve et al., 2016; Hashida et al., 2021)	√ (Stromberg et al., 2016)	×
FGF2	√ (Kumar et al., 2020; Rajendran et al., 2021; Zhu et al., 2021)	×	√ (Homer-Bouthiette et al., 2021; Mathes et al., 2021)	×
LIF	√ (Vela et al., 2016; Bi et al., 2020)	√ (Kapilevich et al., 2017)	√ (Zakharova et al., 2021)	×
Myostatin	√ (Bondulich et al., 2017; Abati et al., 2022)	√ (Allen et al., 2011; Zhou et al., 2021)	√ (Bergen et al., 2015)	×
Myonectin	×	√ (Otaka et al., 2018)	√ (Otaka et al., 2018)	√ (Otaka et al., 2018)
NGF	√ (Eyjolfsdottir et al., 2016)	√ (Arvidsson et al., 2018)	√ (Hayashi et al., 2011)	×
Humanin	√ (Matsuoka and Hashimoto, 2010; Yen et al., 2018)	√ (Woodhead et al., 1985; Gidlund et al., 2016)	√ (Woodhead et al., 1985)	×

CX3CL1 (Fractalkine), CX3C chemokine ligand 1; FGF2, fibroblast growth factor 2; LIF, leukemia inhibitory facto; NGF, nerve growth factor.

### 3.1 BAIBA

After inducing PGC-1α overexpression in the gastrocnemius muscle of mice, a remarkable amplification of over 10-fold was observed in the plasma concentration of BAIBA. Conversely, in PGC-1α knockout mice, the BAIBA levels exhibited an approximate reduction of 23% (Roberts et al., 2014). Furthermore, endurance training increases plasma BAIBA levels in both humans and mice (Roberts et al., 2014). Overexpression of PGC-1α in primary satellite cells of mice induces the secretion of BAIBA, a metabolite of the branched-chain amino acid valine, to 2.7-fold in a serum-free medium (Roberts et al., 2014). Utilizing a frequency of 90 Hz to stimulate Primary cultures of mouse gastrocnemius muscle and soleus muscle, the secretion of BAIBA is observed in the conditioned medium across various cell types. Furthermore, the skeletal muscle of aged mice secreted a more pronounced amount of BAIBA compared to young mice (Kitase et al., 2018).

β-aminoisobutyric acid mitigates the skeletal muscle and bone loss resulting from peripheral disuse. These effects are likely achieved by the regulation of mitochondrial gene expression and the prevention of mitochondrial damage caused by oxidative stress, which leads to apoptosis (Kitase et al., 2018). The anti-inflammatory and anti-insulin resistance effects of BAIBA are confirmed in differentiated 3T3-L1 cells through an AMPK-dependent transduction mechanism. Furthermore, BAIBA promotes fatty acid oxidation in the liver and plays a positive role in adipocytes (Jung et al., 2018). Exercise-induced production of BAIBA attenuates metabolic stress and apoptosis in cardiomyocytes caused by mitochondrial dysfunction, with the involvement of the miR-208B/AMPK pathway (Yu et al., 2022).

β-aminoisobutyric acid exerts central protective effects through its anti-inflammatory properties. After an 8-week period of intraperitoneal BAIBA administration, notable amelioration of hypothalamic inflammation and discernible inhibition of microglial activation was observed in severely obese mice exposed to an extended high-fat diet (Park et al., 2019). A similar anti-inflammatory effect is observed in the BV-2 macrophage cell line treated with BAIBA upon palmitic acid exposure (Park et al., 2019). Furthermore, BAIBA exhibits protective effects against hydrogen peroxide-induced oxidative stress and apoptosis in the pheochromocytoma cell line (PC12) via activation of the AMPK and PI3K/Akt pathway (Minato et al., 2022). The development of NDs is accompanied by oxidative stress, inflammation, and mitochondrial damage, further investigations into the potential of BAIBA in preventing these pathological processes are warranted.

### 3.2 CLU

Apolipoprotein J, also known as CLU, has received extensive attention in the field of NDs due to its widespread expression in the CNS (de Silva et al., 1990). One of the notable roles of CLU is to limit the uptake of extracellular α-synuclein aggregates by astrocytes, thereby promoting the spread of the effects of PD (Filippini et al., 2021). Moreover, CLU plays a crucial role in modulating excitatory synaptic transmission and neuronal dendritic spine density. *In vitro*, supplemented with CLU enhances the frequency of miniature excitatory postsynaptic currents (mEPSCs), then promotes excitatory neurotransmission. More importantly, the expression of CLU in astrocytes is sufficient to rescue the impaired synaptic structure and function in CLU knockout mice and attenuates amyloid pathology and synaptic defects in 5XFAD mice (Chen et al., 2021). Furthermore, peripheral administration of human recombinant CLU regulates the level of Aβ in the brain of APP23 mice, highlighting its potential therapeutic effect (de Retana et al., 2019).

However, the upregulation of CLU leads to the formation of a tau/CLU complex, which enters HEK293T cells through endocytosis. Subsequently, this complex disrupts endolysosomes, which allows the transfer of complex to the cytoplasm and facilitates the translocation of tau protein between cells, which may accelerate tau pathology spread (Yuste-Checa et al., 2021). Compared with AD mice, CLU knockout AD mice (CLU knockout mice crossed with 5XFAD mice) exhibit significantly lower soluble Aβ oligomers and amyloid plaques, as well as higher neuronal and synaptic protein levels. It improved motor coordination and enhanced spatial learning and memory abilities (Oh et al., 2019). However, these beneficial effects of CLU deficiency are observed only at the early stages of AD, and diminished at the age of 10 months, suggesting that CLU participates in the early stages of AD progression (Oh et al., 2019).

Clusterin has been identified as a pivotal factor implicated in enhancing brain health via exercise. Leveraging the impacts of exercise on neuroinflammation and cognitive function, a previous study undertook a comparative analysis of plasma proteomics alterations between individuals who participated in exercise and those in a non-exercise cohort. Among the proteins scrutinized, four, including CLU, demonstrated the most prominent distinctions. Subsequently, the four proteins in the plasma of individuals with exercise are neutralized and injected into the neuroinflammation model induced by LPS (Jeon et al., 2020). The results show that inhibition of CLU substantially abolishes the anti-inflammatory effect in plasma with exercise, while inhibition of the other three proteins has no effect (Jeon et al., 2020). Nevertheless, the source of the CLU secreted in circulation during exercise is still unclear. CLU is expressed in various tissues and organs, with the liver being the primary peripheral source of this protein (Seo et al., 2020). It is demonstrated that muscles can express CLU (Kropackova et al., 2021), but further genetic validation is necessary to determine whether CLU can function as a myokine produced during exercise and serve as a critical factor in improving NDs.

## 4 Discussion

In conclusion, the emerging exerkines, which are previously unappreciated signaling molecules secreted by skeletal muscle during exercise, hold significant promise in the regulation and treatment of NDs. Through their anti-oxidative stress, anti-inflammatory, and mitochondrial-enhancing properties, exerkines have shown the potential in improving brain health and ameliorating the pathogenesis of NDs. This review highlighted several keys previously unappreciated exerkines, including apelin, lactate, KYNA, BAIBA, and CLU, especially their roles in modulating neuroinflammation, synaptic plasticity, mitochondrial function, and overall neuroprotection.

The findings emphasize the importance of exploring the function and mechanisms of exerkines in NDs. Further investigation is warranted to elucidate the specific signaling pathways and underlying molecular mechanisms through which exerkines exert their neuroprotective effects. Understanding the mechanisms that receptors of exerkines and intracellular pathways are involved in signaling will provide valuable insights into their therapeutic potential. Additionally, optimizing exercise protocols to enhance exerkines secretion and release will be important for maximizing their therapeutic potential. Taking lactate as an example, due to the direct causal relationship between exercise intensity and lactate production, researchers should consider the interactive effects between increased lactate levels resulting from physical activity and the negative impacts of overtraining. Studies indicate that overtraining can induce a spectrum of detrimental changes, known as overtraining syndrome (Kreher and Schwartz, 2012; Carrard et al., 2022). These changes may manifest specifically as hormonal imbalances (McEwen, 2020), increased oxidative stress and inflammation (Jelinek et al., 2021), disruption in neurotransmitter equilibrium (Snowden et al., 2019), cellular apoptosis (Singh et al., 2019), and acidosis (Li et al., 2010). Such alterations significantly impair brain health. The existence of the BBB implies that the brain’s uptake of lactate does not increase indefinitely with the rise in systemic lactate levels but maintains a relatively stable state. This regulation is closely linked to the phosphatase and tensin homolog (PTEN)/Akt/MCT1 signaling pathway in cerebral vascular endothelial cells. However, excessively high intracerebral lactate levels may cause abnormalities in adult hippocampal neurons, subsequently affecting hippocampus-dependent learning and memory functions (Wang et al., 2019). Therefore, to maximize the positive effects of physical exercise, it is crucial to implement a balanced exercise regimen.

Overall, the function of previously unappreciated exerkines represents a promising area of research in the field of NDs. For instance, the infusion of sodium lactate did not enhance semantic categorization processes in AD patients (Kalman et al., 2005a), yet it altered cortical blood flow dynamics in these individuals (Kalman et al., 2005b). Although the clinical application of these exercise-related factors remains relatively underexplored, the utilization of analogs, such as SZR104 and KYNA-amide, analogs of KYNA, provides novel perspectives in clinical research. The latter has demonstrated potential in improving learning and memory capabilities in animal models (Gellert et al., 2012). Continued investigation into the specific roles and mechanisms of these exerkines may lead to the development of innovative treatments and interventions that can improve the lives of individuals affected by NDs.

From the effects of several factors discussed, we can conclude that anti-oxidative stress and anti-inflammation are essential mechanisms for the function of myokines, as well as the improvement of mitochondrial function by transcriptional regulation. For example, upon activation, PGC-1α regulates the expression of KATs and increases the expression and release of irisin precursor FNDC5. Intriguingly, apelin can regulate PGC-1α. The crosstalk and the synergy between different myokines are also worthy of future investigation, which will help clarify the complex beneficial effect on ND progression of exercise.

At present, the discussion of exerkines in NDs mainly focuses on verifying their “existence.” In addition, the difference in exercise prescription greatly affected the research results. This review involved data acquired at different times, intensities, and forms of exercise, but these different aspects may bring about similar results. Is it due to the susceptibility of exerkines in response to exercise? The low responsiveness to exercise stimuli signifies that different types of exercise may produce different concentrations or types of myokines. Furthermore, despite the scarcity of studies, an extrapolation from the presented tables suggests that the response to physical activity might vary not only across athletic cohorts of distinct ages, but also among various types of muscle fibers (Tables 2, 3). The answers to these questions have important implications for explaining the multiple effects of different forms of exercise. On the contrary, clarifying the mechanism of myokine action enables the specific and targeted formulation of exercise prescription. It thus supports the diagnosis and prescription of NDs.

In addition to skeletal muscle, the adaptation of various body organs elicited by exercise is significant as well. More myokines may indirectly affect brain health by connecting the skeletal muscle with various organs to trigger downstream cascading effects. Collectively, the motor system is presumed to initiate the whole process. Therefore, it is necessary to verify the existence and function of myokine in the improvement of exercise-induced ND patients from a genetic point of view.

## Author contributions

XB: Writing – original draft. QW: Writing – review and editing. YW: Writing – review and editing. SL: Writing – review and editing, Supervision.
